# A Finite Element Study of Simulated Fusion in an L4-L5 Model: Influence of the Combination of Materials in the Screw-and-Rod Fixation System on Reproducing Natural Bone Behavior

**DOI:** 10.3390/biomimetics10020072

**Published:** 2025-01-24

**Authors:** Mario Ceddia, Luciano Lamberti, Bartolomeo Trentadue

**Affiliations:** Department of Mechanics, Mathematics and Management, Polytechnic of Bari, 70125 Bari, Italy; bartolomeo.trentadue@poliba.it

**Keywords:** spinal fusion surgery, biomimetic materials, PEEK, FEA

## Abstract

The mechanical properties of materials for spinal fixation can significantly affect spinal surgical outcomes. Traditional materials such as titanium exhibit high stiffness, which can lead to stress shielding and adjacent segment degeneration. This study investigates the biomechanical performance of titanium and PEEK (polyetheretherketone) in spinal fixation using finite element analysis, through the evaluation of the *Shielding Strength Factor* (*SSF*). **Methods:** A three-dimensional finite element analysis (FEA) model of an L4/L5 functional spinal unit was developed to simulate the mechanical behavior of three fixation systems: titanium screws and rods (model A), titanium screws with PEEK rods (model B), and PEEK screws and rods (model C). The analysis evaluated stress distribution and load transfer under physiological conditions, in comparison with the intact spine (baseline model). **Results**: The analysis showed that titanium fixation systems resulted in higher stress shielding effects, with a significant difference in stress distribution compared to PEEK. The maximum stress recorded in the neutral position was 24.145 MPa for PEEK, indicating better biomechanical compatibility. **Conclusions**: The results suggest that PEEK may be an attractive alternative to titanium for spinal fixation, promoting more healthy load transfer and minimizing the risk of stress shielding complications.

## 1. Introduction

Spinal fusion surgery is performed to stabilize the vertebral column in cases involving degeneration, deformity, fractures, and intervertebral disk diseases, or following tumor excision. In the United States, approximately 350,000 spinal fusion procedures are conducted annually [1]. This number is expected to rise, primarily due to the aging population and an increase in degenerative conditions, including obesity. The technique involves the insertion of pedicle screws into adjacent vertebrae to stabilize them [1,2,3,4]. These screws are connected by metal rods that provide structural support. It has been observed that adequate screw fixation is crucial, as inadequate fixation can lead to complications such as screw loosening or breakage [5,6,7,8]. One study indicated that 4.7% of 658 screws inserted had loosened, and 0.46% had broken within three and a half years of insertion [9].

Metallic materials such as titanium alloys are used in rigid fixators, which primarily support the load, thereby reducing the load experienced by the vertebral segments and resulting in a phenomenon known as “stress shielding”. Consequently, the fused segment may weaken if it does not receive sufficient load to promote its health and strengthening. This issue arises from the difference in stiffness between the implant and the surrounding bone. For example, the elastic modulus of cortical bone typically ranges from 1 to 20 GPa, whereas titanium has an elastic modulus of approximately 110 GPa [10,11]. This significant difference inhibits effective load transfer to the bone, resulting in stress shielding that prevents the bone from receiving the mechanical stimuli necessary to maintain its integrity. Additionally, the high rigidity of titanium fixation systems can disrupt normal spinal load and movement, causing excessive stress on adjacent vertebral segments, which may accelerate their degeneration compared to a healthy, flexible spine.

Several researchers have studied the phenomenon of stress shielding (SS) [12,13,14,15,16,17,18]. Meena et al. [19] analyzed the stresses and deformations in porous implants under various loading conditions using the finite element method (FEM). The results showed that the reduction in stress shielding was primarily due to pore size, which ranged from 0.4 to 0.6 mm, suggesting that pore size has a significant impact on the structure of the implant. Tsuang et al. [20] designed a biomimetic porous implant and conducted simulations and mechanical tests to study the stresses and deformations. They concluded that heat treatment applied to the implant improved its material properties, optimizing the stiffness of the device, thereby reducing stress shielding and providing adequate space for bone growth. Additionally, biomimetic materials can improve load sharing between the bone structures and the fixation system [21,22,23,24]. This can minimize the effect of stress shielding should the load be primarily transferred to the fixation device rather than the bone, thereby promoting better integration and bone fusion. Currently, the adoption of polymeric materials in spinal surgery represents a promising approach to improve clinical outcomes, reduce complications, and promote more effective bone fusion [25,26,27,28,29]. For example, polymer materials such as polyetheretherketone (PEEK) have a much lower modulus of elasticity (around 3.6 GPa) than titanium (110 GPa). This reduces the stiffness of the fixation system, allowing a better load distribution, which is more like that of a natural spine [23,24]. In a study conducted by Becker et al. [30], it was observed that hybrid screws made of carbon fiber composite and PEEK led to a reduction in stress shielding compared to titanium screws. However, it emerged that bone quality plays a crucial role in the transmission of stress between the implant and the bone. Specifically, less dense bone resulted in greater mobility of the PEEK screws, increasing the risk of fixation loss. In a numerical study conducted by Eastlack et al. [31] it was found that vertebral fixation between the L4 and S1 levels using PEEK screws provided comparable stability to that of standard posterior fixation systems. From a biomechanical perspective, it was observed that the PEEK screws exhibited comparable performance to titanium screws in terms of fatigue resistance [32,33,34,35].

Recently, dynamic and flexible instrumentation systems, such as the Dynesys system, as well as semi-rigid systems like the PEEK rod–pedicle screw system, have been introduced as alternative biomaterials for spinal support [32]. Biomechanical studies have demonstrated that PEEK rods provide enhanced durability, stability, strength, and an overall superior biomechanical profile compared to metallic rod systems [32,33,34,35,36,37,38,39,40,41].

Nowadays, finite element analysis (FEA) offers a distinct advantage over clinical studies, as it allows researchers to simulate and analyze stress, strain, and displacement patterns within the spine and implant components. This method enables precise control over simulation parameters, facilitating the investigation of potential causes of implant failure, loosening, and complications that are challenging to assess clinically. It is well known that numerous finite element studies have been carried out to compare the effectiveness of PEEK with that of metallic materials such as titanium [35,36,37,38,39,40,41]. However, in the literature, there are few studies that have numerically evaluated, with the finite element method, the effect of stress shielding in intervertebral fixation prostheses. The purpose of this study is to provide a biomechanical understanding of the use of three fixation systems (rigid and semi-rigid) using titanium and PEEK pedicle screws and rods, using the FEM. Three models of spinal fixation are compared with the intact (baseline) model: model A, with screws and rods made of titanium; model B, with titanium screws and PEEK rods; and model C, made entirely of PEEK. In particular, the effect of stress shielding is evaluated by comparing stress values between the intact L4-L5 segment and the prosthetic models. The novelty of this study is in its use of an all-PEEK fixation system. The hypothesis analyzed here is that the use of a fixation system made entirely of PEEK will reduce the problem of stress shielding and guarantee the same mechanical stability as that achieved by fixation systems made of titanium.

## 2. Materials and Methods

The analysis of the biomimetic behavior of a PEEK-based spine fixation system was conducted using the finite element method (FEM). For this purpose, the real object has to be simplified to create a numerical model. In this study, a patient-specific model was generated, consisting of two vertebrae in the L4-L5 segment [33,34]. This functional spinal unit (FSU) was chosen because clinical studies have shown that the L4-L5 level is one of the levels that is most affected by lumbar degenerative disease [42,43,44]. Furthermore, epidemiological studies have highlighted that the L4-L5 level exhibits a higher incidence of disk herniation and spinal stenosis compared to other lumbar levels, making it a focal point for research on spinal fusion and fixation materials [45].

**Figure 1:**
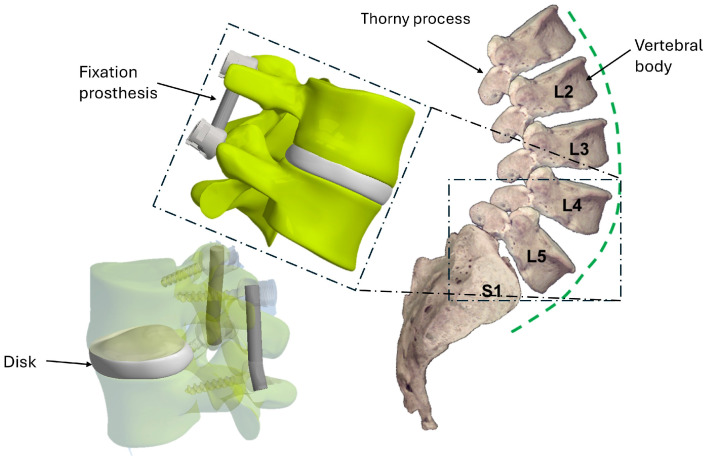
A schematization of the two-vertebrae system, L4-L5,analyzed in this study.

The model analyzed in the study refers to a specific 30-year-old male patient, but the results obtained may have general validity. Using the 3D CAD software Autodesk Inventor 2024, the vertebrae were assembled by inserting an intervertebral disk between them, as shown in Figure 1.

**Figure 2:**
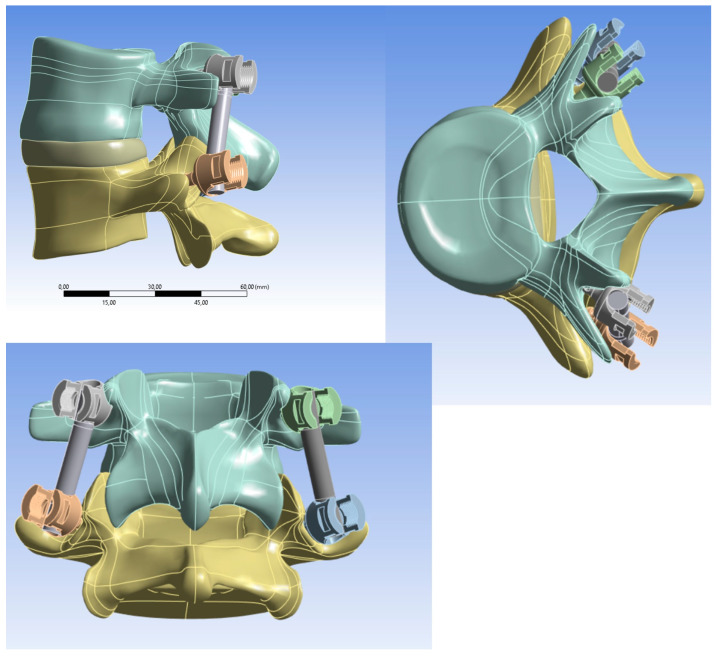
Construction of ANSYS model of L4-L5 vertebrae complex with pedicle screws. The “,” notation in the size bar of the first figure indicates the decimal signs.

Once the two vertebrae were assembled, the model was saved in STL format. Subsequently, the geometry was imported into Autodesk Meshmixer 3.3.15 to convert the surface mesh into solid elements and save the model in STP format. Finally, the geometry was imported into SpaceClaim R2023 to separate the vertebrae and insert the pedicle screws. The FEM model was reviewed and processed in the finite element software ANSYS R2023. At this stage, the geometry was imported into ANSYS, where the necessary working surfaces for defining the model were created in the Part module, as shown in Figure 2.

### 2.1. Modeling of Material Mechanical Properties

The mechanical properties of bone vary in relation to the type of bone tissue considered (cortical and trabecular bone), the physical characteristics of the individual, and the methods used to perform mechanical tests [46,47,48]. This leads to significant variability in the mechanical properties reported in the literature. Currently, there is a considerable dispersion of data regarding the mechanical properties of both trabecular and cortical bone, as the configuration of trabeculae varies among different types of bone, and even within the same bone. Therefore, to define the mechanical properties of bone tissue, the relationship between apparent density and elastic modulus (*E*) was utilized, see Equation (1). For each simulation, homogeneous values of *E* were assigned throughout the vertebra, treating it as an isotropic material [49].

(1) $$E=4750\rho^{1.56}$$

The intervertebral disk has two main components: the annulus fibrosus (AF) and the nucleus pulposus (NP). The fibrous annulus consists of concentric layers of collagen fibers that provide tensile strength, while the nucleus pulposus is a gelatinous substance that accounts for compressibility. From a mechanical point of view, the disk exhibits hyperelastic behavior, which is characterized by its ability to undergo large deformations and return to its original shape when the load is removed [50]. However, the modeling of a hyperelastic component requires numerous parameters that are difficult to find in the literature. The mechanical tests performed by Yang et al. [51] allowed us to measure the linear elastic modulus of the entire disk, which is treated as an elastic and homogeneous material. This approach made it possible to simplify the complex behavior of the real disk, while providing useful values for the numerical implementation of its behavior in finite element models. Regarding the materials of the prosthesis (screws, rods), linear elastic and homogeneous behaviors were assumed. The data were sourced from the literature, and are presented in Table 1 [49,51,52].

**Figure 3:**
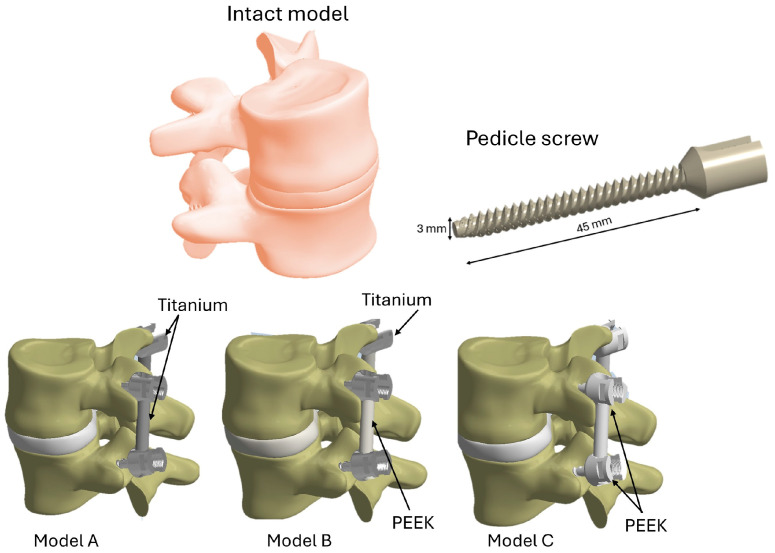
Three-dimensional models of spine fixation and intact spinal segment analyzed in this study.

The analysis involved comparing two materials (PEEK and Titanium alloy) used in spinal surgery with the intact (baseline) model. In the first model (Model A), titanium was used for the pedicle screws and fixation rods. In the second model (Model B), titanium was used for the pedicle screws and PEEK for the fixation rods. Finally, in the third model (Model C), both the screws and rods were modeled using PEEK as the common material. The pedicle screw chosen had a length of 45 mm and a diameter of 3 mm, with a single thread, as shown in Figure 3.

### 2.2. Modeling of Loads and Constraints

**Figure 4:**
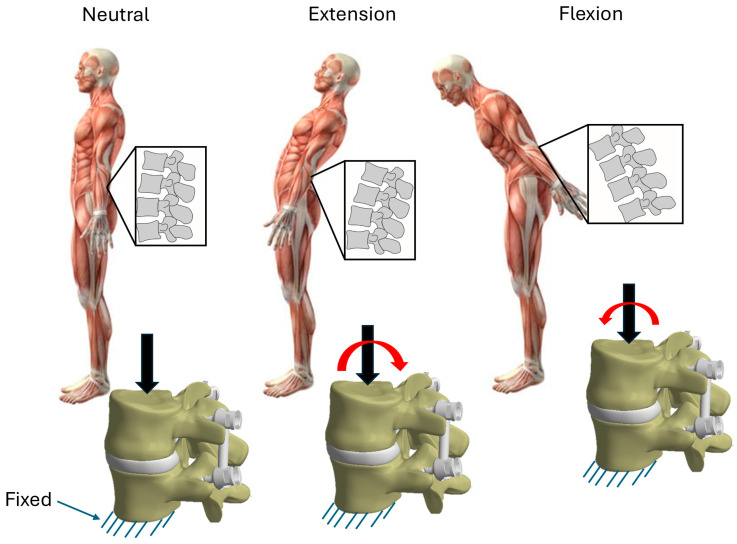
Loads and constraint conditions in various positions. Vertical force and bending moment are denoted by the black arrow and the read arrow, respectively.

The loads applied to the two vertebrae correspond to the phases in which a person is in the neutral position, in extension and in flexion. Rotational loads should also be considered, but they have a lower intensity than loads in the previously defined positions, as shown by some FEA studies [52,53,54]. Therefore, only the loading modes shown in Figure 4 were considered in the comparative analysis performed here. These conditions correspond to a 30-year-old person weighing 95 kg. Table 2 shows the numerical values of the loads assumed in the three phases, applied to the upper surface of the L4 vertebra. Meanwhile, the lower part of the L5 vertebra was constrained in all directions [49,55].

Fixed contact conditions were assigned between the screws and the vertebrae. Frictional contact was assumed between the screws and the fixation rods, with a coefficient of friction of 0.3.

### 2.3. Finite Element Modeling and Analysis

**Figure 5:**
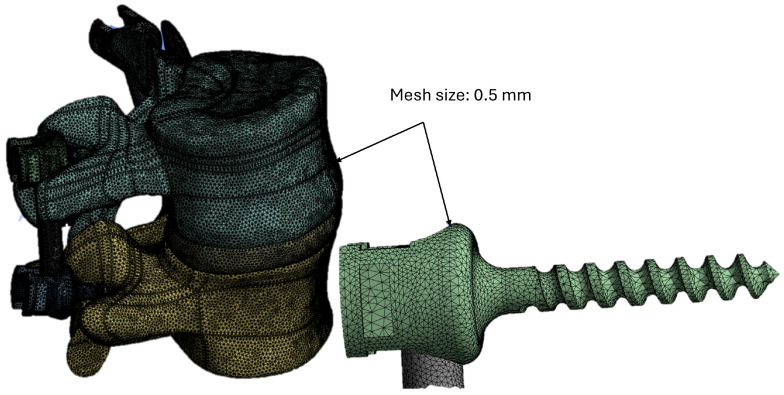
The finite element model of the L4-L5 vertebrae and the implant.

After assigning the material properties and the loading and boundary conditions, the 3D model of the fixed L4-L5 assembly was converted into finite elements. Given the complexity of the geometry, tetrahedral elements were selected, which ensure a higher level of accuracy compared to hexahedral elements. To ensure the accuracy of the results obtained from the finite element analysis (FEA), a mesh convergence study was conducted, setting an acceptable percentage variation of 1% between the results of successive meshes. Three different meshes were hence created, including 100,000, 280,000, and 300,000 elements, respectively. The von Mises stress distributions computed for each mesh were compared. Since the largest stress variation between the 280,000 element and the 300,000 element meshes was less than 1%, convergence was considered to be achieved. Consequently, a final mesh including 289,456 elements was selected for the analysis, utilizing a mesh size of 0.5 mm. This approach is consistent with findings reported in the literature [56,57,58,59,60]. The finite element model of the L4-L5 vertebrae and the implant is shown in Figure 5.

The von Mises criterion selected for stress evaluation assumes that a material begins to yield when the equivalent stress reaches a certain critical value, which is related to the tensile strength of the material itself. Specifically, the von Mises criterion is based on the idea that the failure of a material occurs not only due to normal stresses (stresses acting perpendicular to the surface), but also due to shear stresses (stresses acting parallel to the surface).

### 2.4. Validation of the FE Model

The finite element model developed in this study for the fixed L4-L5 spine segment was validated by comparing the Von Mises stress distributions in the vertebrae computed for the three fixation models and for the intact (baseline) model. The validation process also relied on studies previously published in the literature [49,52,53,54,55] which evaluated the stresses transmitted by the prosthesis on the FSU; remarkably, the results obtained in this study showed good consistency with those reported in the literature.

## 3. Results

**Figure 6:**
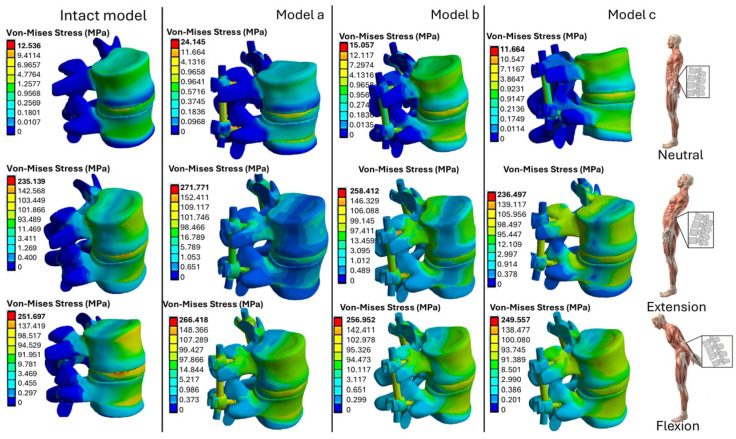
The von Mises stress distributions computed by ANSYS for the fixed L4-L5 models and the intact (baseline) model analyzed in this study.

The numerical results for the stress distribution in the L4–L5 vertebrae, intervertebral disk, and prosthetic devices, such as the pedicle bar and pedicle screws, are shown in Figure 6. The stress values in three different positions (neutral, flexion, and extension) for the three models (A, B, C) were compared with their counterparts for the intact (baseline) model.

Compared to the neutral position, the results show an increase in stress during flexion and extension. Model A, with titanium rods and pedicle screws, shows the highest stress values, while Model C, with PEEK components, shows the lowest stress level (11.664 MPa in neutral position). In extension, Model A reaches 271.771 MPa, compared to 24.145 MPa recorded in neutral position. This indicates that the inclination of the body results in insignificant stresses, due to a bending moment, relative to the neutral position. In addition, the use of polymeric materials such as PEEK reduces stress concentration areas and improves stress distribution between the vertebrae and the prosthesis.

### Stress Shielding Evaluation

**Figure 7:**
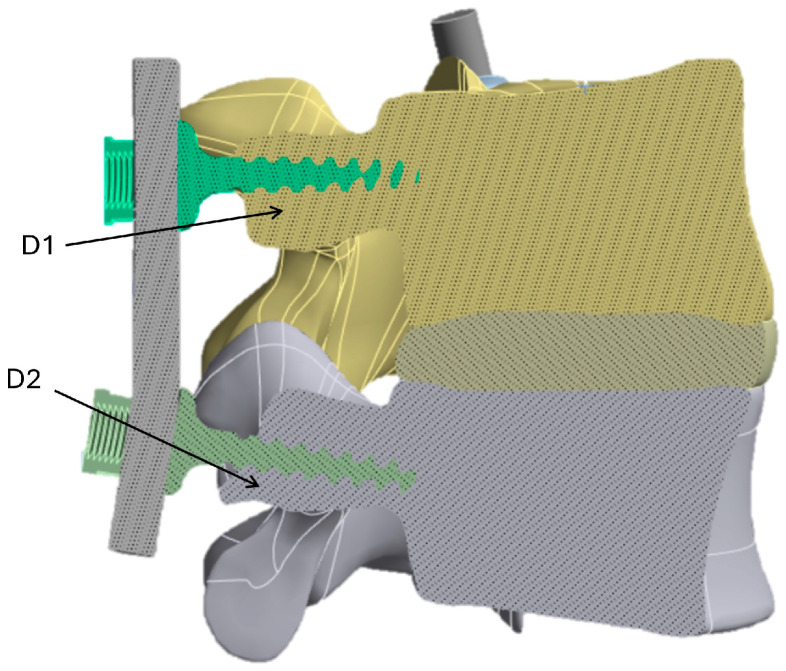
D1 and D2 control locations for evaluating stress shielding factor.

An implant that stresses the bone structures differently than the intact model may cause stress shielding and bone density loss, because the bone near the pedicle screw, where the highest stresses are exchanged, is not stimulated, and hence may resorb. In contrast, an implant that distributes stresses more evenly may promote bone growth at the screw surface and full osteointegration. To analyze stress shielding, the representative control locations (D1 and D2) near the bone/screw interface, where stress shielding is most likely to occur, were considered (see Figure 7).

To define the stress shielding factor (*SSF*) in %, the average stress $\left(\sigma_{intact}^{M}\right)$ was calculated, considering the control points D1 and D2 of the intact model shown in Figure 7, and was compared with its counterparts $(\sigma_{implant}^{M}$) for the three prosthetic models analyzed in this study, using Equation (2):

(2) $$Stress\;shielding\;factor\;\left(SSF\right)=\frac{\sigma_{intact}^{M}-\sigma_{implant}^{M}}{\sigma_{intact}^{M}}\times 100$$

A positive *SSF* value indicates that the stress developed at the control locations is lower in the prosthesis model than in the intact (baseline) model. A negative *SSF* value indicates that stress is lower in the intact model.

**Figure 8:**
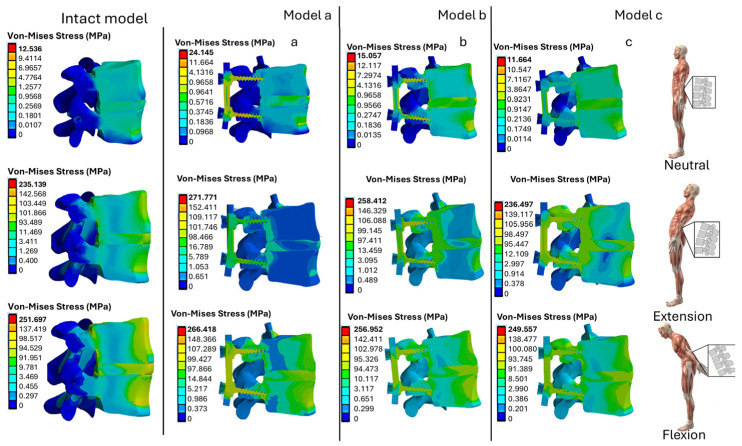
Section view of von Mises stress distributions computed by ANSYS for L4-L5 fixation models A, B, and C, and for intact (baseline) model, analyzed in this study. Subfigures (**a**–**c**) correspond to models A, B, and C, respectively.

Figure 8 shows the section view of the stress distributions computed for the fixation models A, B, and C and for the intact (baseline) model. Using the results obtained in Figure 8, it was possible to evaluate the *SSF* values, which are listed in Table 3.

It can be seen from Table 3 that in the case of model A, for all positions, the value of *SSF* varies between 1.45% and 61.4%, indicating a slight stress shielding phenomenon in flexion, and a much more relevant effect in the neutral and extension positions. In the case of model B, stress shielding is observed in all positions, with a negative peak *SSF* value of −166.5% in the flexion position. Using an all-PEEK prosthesis (model C) significantly increases stress at control locations D1 and D2 compared to the intact (baseline) model, thus promoting bone growth and limiting the stress shielding problem. In fact, negative *SSF* values are observed in this case, ranging from −1181.3% in the neutral position to −339.8% in flexion.

**Figure 9:**
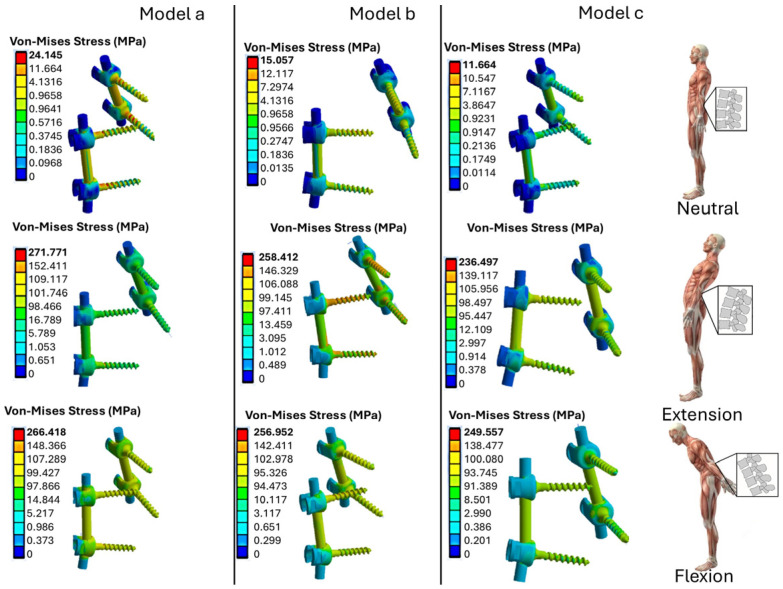
Detailed von Mises stress distributions computed by ANSYS for the screws and rods of the three fixation models analyzed in this study.

Figure 9 shows the stress values computed for on the pedicle screws and rods for the three fixation models. In Model A, in the extended position, the highest stresses are found at the beginning of the screw thread. On the other hand, in model C, in the neutral position, the lowest stress values are observed on the screws. The PEEK rods are more stressed than the titanium ones in both extension and bending. In fact, the stress values for the PEEK bars vary between 100 MPa and 110 MPa, while values between 92 MPa and 96 MPa are found for the titanium rods.

From the results obtained, Model A absorbs most of the stresses, limiting transmission to the bone and generating high stress concentrations. The all-PEEK solution (Model C) distributes stresses more evenly between the prosthesis, vertebra, and disk, reducing stress shielding and promoting better healing.

## 4. Discussion

Postoperative complications of posterior interbody fusion (PLIF), such as adjacent segment degeneration (ASD), increase the probability of revision surgery [61,62,63]. The use of spinal instrumentation with titanium pedicle screws and rods provides rigid stabilization of the spine due to the high stiffness of this material. This leads to high fusion rates and satisfactory long-term clinical results [64,65,66]. However, higher implant stiffness is associated with a lower capacity to absorb loading forces, resulting in inadequate load distribution at the screw–bone interface. In addition, rigid implants can shift the load away from the bone elements, leading to a shielding effect. This effect reduces bone density and can compromise implant anchorage.

To overcome the disadvantages of titanium devices due to their high stiffness, less rigid implants made of PEEK have been developed. Biomechanical studies analyzing the properties of fixation with PEEK and titanium rods have reported similar spinal stability for the two materials [67,68,69,70]. Wang et al. [53] found that PEEK rods transferred less stress to adjacent segments. In a comparative biomechanical study by Li et al. [52], it was found that PEEK rods reduced the stress on the screw–rod system by approximately 50% compared to titanium. In addition, the use of PEEK pedicle screws showed benefits, particularly in reducing the risk of screw loosening, as shown in the analysis by Oikonomidis et al. [71]. Clinically, PEEK has also led to significant improvements. For example, in a retrospective study by Ho et al. [72], the results indicated that PEEK pedicle screws significantly improved postoperative CT imaging and reduced artifact indices compared to titanium. Qi et al. [73] compared 20 patients with PEEK rods and 21 patients with titanium rods, and found no significant differences in fusion rates or clinical outcomes. However, the effect of stress shielding was not evaluated by comparing different models in most of the studies found in the literature. In order to fill this gap, the aim of this study was to compare three interbody fixation systems. Specifically, in Model A, both the screws and rods were made of titanium, while in Model B, the screws were made of titanium and the rods were made of PEEK. For Model C, PEEK was considered for both components. The analysis, performed using the finite element method, evaluated the effect of stress shielding from a numerical perspective, but neither in vivo nor in vitro.

The FEA results indicated that high stress values occurred in the flexion position for the A model, with peaks of up to 87 MPa. This highlights how titanium significantly concentrates stresses, particularly at the beginning of the threading of the pedicle screws. In addition, the high stiffness of titanium absorbs most of the stresses, limiting load transfer to the bone and resulting in high stress concentrations. In fact, this model was found to have a maximum stress shielding factor (*SSF*) of 61.4% in the extension position. Model B combined titanium elements with PEEK components, taking advantage of the benefits of both materials. However, high stress regions can be localized at the PEEK/titanium transition regions. No stress shielding was observed for this fixation model, but the load transferred to the bone in the extension position was rather low (*SSF* is only −29.4%). Model C showed a much more homogeneous stress distribution, improved bone integration, and no evidence of stress shielding in any loading mode. Notably, the *SSF* was between about 2 times and about 30 times lower than that for Model B. The better stress distribution achieved by model C, with respect to the other fixation models A and B, would certainly help to achieve a complete fusion of the L4-L5 spine segment considered in this study.

The lower stiffness of PEEK allows for better adaptability to load changes, reducing the risk of fracture or device failure [71]. This study showed, in agreement with the literature, that the contact zone between the screw and bone is prone to bone tissue failure [49,74,75,76], as the highest stresses were reached in this area. In addition, the results of this study showed that the flexion position increases the load on the vertebral segments, as forward flexion of the spine increases the pressure on the intervertebral disks and anterior structures, as clearly visualized in Figure 7.

### Limitations

The present study has several limitations. Firstly, only one FSU was used for the L4-L5 segments, rather than the entire spine. To provide a qualitative comparison of the effectiveness of the use of PEEK in interbody fixation methods, the aim of this study was to evaluate the effect of stress shielding in the most stressed FSU. Future studies will consider the different biomechanical interactions between individual vertebrae. In addition, the data were obtained from a 30-year-old healthy male subject, but the human spine varies according to age, disease, and other factors. This could alter the results, due to the variety of spinal conditions (such as severe disk degeneration). The analysis of this study focused on evaluating the stresses transmitted between the prosthetic components and the vertebrae, without considering the surgical approach used in lumbar fusion and tissue preservation. Therefore, ligamentous structures were not considered. Future analysis will improve the modeling as it is implemented. However, since the above-mentioned limitations occur for all models, they do not affect the relative behavior of models A, B, and C.

In terms of materials and prosthetic components, the screw was molded in two materials, titanium and PEEK. While there are clinical studies in the literature that have also investigated composite materials, such as carbon fiber-reinforced PEEK coated with osteoinductive materials [67,68,69,70,71,72,73,74,75], the main goal of this study was to study a full-PEEK fixation structure. The assumption of quasi-static loading made in this study is another significant limitation, as dynamic loading stresses the prosthetic components. However, dynamic loading was not considered, due to the lack of accurate experimental data on dynamic loading [37,49,68]. Despite the limitations, the finite element (FE) model showed good agreement with existing results, and was validated by comparison with stress trends from previous numerical work. Although differences in material geometry and properties could affect the absolute stress values, the overall trends remained consistent.

## 5. Conclusions

This study provided significant evidence for the use of polyetheretherketone (PEEK) in vertebral fixation, suggesting that it may be a promising alternative to traditional materials such as titanium, with potential biomechanical and clinical advantages. The use of polymeric materials such as PEEK in spinal surgery can not only optimize biomechanical outcomes, but also promote more effective bone fusion, helping to mitigate the risks associated with hyperstabilization and stress shielding. However, it is important to note that most of the currently available studies have limited follow-up and involve relatively small sample sizes, which limits the ability to draw definitive conclusions regarding the long-term benefits and potential complications associated with the use of PEEK. Therefore, this biomechanical study provides a solid baseline for future investigations that could further demonstrate the efficacy and safety of PEEK in the clinical practice of spinal fusion.

## Figures and Tables

**Table 1:** Material properties used in finite element analysis of lumbar spine.

Material	Young’s Modulus [MPa]	Poisson’s Ratio
Vertebrae L4-L5	11,000	0.3
Disk	25	0.45
PEEK *	3600	0.25
Titanium alloy (Ti-6Al-4V)	110,000	0.3

* PEEK stands for polyetheretherketone.

**Table 2:** Summary of load simulations for 30-year-old man weighing 95 kg [55].

Position	Vertical Force [N]	Bending [Nm]
Neutral	486	0
Extension	486	76.5
Flexion	486	95.3

**Table 3:** The average values of the von Mises stress and the corresponding values of the stress shielding factor (*SSF*), calculated by averaging the stresses at the representative control locations D1 and D2.

	Von Mises Stress at Control Locations D1 and D2 (MPa)				*SSF* (%)		
	Intact Model	Model A	Model B	Model C	Model A	Model B	Model C
**Neutral**	0.658	0.345	0.934	8.431	47.5	−41.9	−1181.3
**Extension**	6.425	2.481	8.313	9.568	61.4	−29.4	−48.9
**Flexion**	3.514	3.463	9.364	15.455	1.45	−166.5	−339.8

## Data Availability

All experimental data to support the findings of this study are available upon request by contacting the corresponding author.
